# Prof’s Cook and Rogers

**Published:** 1848-04

**Authors:** 


### PROF'S. COOK AND ROGERS.

These gentlemen, who, within the last few months, have both retired from the school, deserve much from the profession at large. Since the first efforts at an organization they have labored assiduously and ardently for the advancement of the school and of dental science. This, too, has been at no small sacrifice of time and money. We are sorry that circumstances have turned up so as to require of them the discontinuance of their labors with us.—CIN. ED.

